# Rationale and design of a prospective study: Cervical Dystonia Patient Registry for Observation of OnaBotulinumtoxinA Efficacy (CD PROBE)

**DOI:** 10.1186/1471-2377-11-140

**Published:** 2011-11-04

**Authors:** Joseph Jankovic, Charles H Adler, P David Charles, Cynthia Comella, Mark Stacy, Marc Schwartz, Susan M Sutch, Mitchell F Brin, Spyridon Papapetropoulos

**Affiliations:** 1 Baylor College of Medicine, Department of Neurology, Houston, TX, USA; 2 Mayo Clinic, Department of Neurology, Scottsdale, AZ, USA; 3 Vanderbilt University Medical Center, Department of Neurology, Nashville, TN, USA; 4 Rush University Medical Center, Department of Neurological Sciences, Chicago, IL, USA; 5 Duke University Medical Center, Department of Neurology, Durham, NC, USA; 6 MedNet Solutions, Inc., Biostatistics, Minnetonka, MN, USA; 7 UBC-Envision Group, Scientific Solutions, Philadelphia, PA, USA; 8 Allergan, Inc., Global Drug Development, Irvine, CA, USA; 9 University of California, School of Medicine, Irvine, CA, USA; 10 Allergan, Inc., Medical Affairs, Irvine, CA, USA; 11 University of Miami, Miller School of Medicine, Miami, FL, USA

## Abstract

**Background:**

A registry of patients with cervical dystonia (Cervical Dystonia Patient Registry for Observation of onaBotulinumtoxinA Efficacy [CD PROBE]) was initiated to capture data regarding physician practices and patient outcomes with onabotulinumtoxinA (BOTOX^®^, Allergan, Inc., Irvine, CA, USA). Methods and baseline demographics from an interim analysis are provided.

**Methods/Design:**

This is a prospective, multicenter, clinical registry in the United States enrolling subjects with cervical dystonia (CD) who are toxin naïve and/or new to the physicians' practices, or who had been in a clinical trial but received their last injection ≥ 16 weeks prior to enrollment. Subjects are followed over 3 injection cycles of onabotulinumtoxinA, with assessments at time of injection and 4-6 weeks later. Information on physician's practice, patient demographics, CD disease history, duration of treatment intervals and neurotoxin dose, dilution, use of electromyography, and muscles injected are collected. Outcomes are assessed by physicians and subjects using various questionnaires.

**Discussion:**

This ongoing registry includes 609 subjects with the following baseline data: 75.9% female, 93.6% Caucasian, mean age 57.6 ± 14.3, age at symptom onset 48.3 ± 16.2, and time to diagnosis 5.4 ± 8.6 years, with an additional 1.0 ± 3.5 years before treatment. Of those employed at the time of diagnosis, 36.6% stopped working as a result of CD. CD PROBE, the largest clinical registry of CD treatment, will provide useful data on current treatment practices with onabotulinumtoxinA, potentially leading to refinements for optimization of outcomes.

**Trial registration:**

NCT00836017

## Background

Cervical dystonia (CD), the most common form of adult-onset focal dystonia, is manifested by sustained, involuntary contractions of the cervical musculature [1]. Patients usually present with pain and postural changes of the neck, often associated with irregular head tremor (dystonic tremor) [2]. Impaired neck mobility, chronic pain, and a reduction in the patient's self-image may adversely impact quality of life and result in disability [3,4].

Physicians and patients had few treatment options prior to the introduction of botulinum toxin for CD over a quarter century ago [5]. Supported with evidence from randomized controlled trials and meta-analyses, botulinum toxin has become the treatment of choice for CD [6,7]. An evidence-based review by the Therapeutics and Technology Assessment Subcommittee of the American Academy of Neurology concluded that botulinum toxin should be offered as a treatment option to patients with CD, a level A recommendation [8]. Botulinum toxin exerts its effect by inhibiting the presynaptic release of acetylcholine from peripheral terminals of motor neurons, thus causing chemodenervation and weakness of the injected muscle [9].

Four botulinum toxin products are approved for CD. Three are serotype A (onabotulinumtoxinA [BOTOX^®^, Allergan, Inc., Irvine, CA, USA]; abobotulinumtoxinA [Dysport^®^, Ipsen, Paris, France]; incobotulinumtoxinA [Xeomin^®^, Merz Pharmaceuticals GmbH, Frankfurt, Germany]) and one is serotype B (rimabotulinumtoxinB [Myobloc^®^/Neurobloc^®^, Solstice Neurosciences, San Francisco, CA, USA]). Each differs in molecular structure, formulation, and clinical profiles. There is no international potency reference standard for botulinum toxins and each formulation of botulinum toxin is different. Therefore, the units of activity are specific to each product and not interchangeable with those of any other botulinum toxins [10].

The objective of this paper is to describe the rationale, study design, and baseline characteristics of patients participating in Cervical Dystonia Patient Registry for Observation of onabotulinumtoxinA Efficacy (CD PROBE; NCT00836017), a large observational study designed to capture data on the clinical presentation, dosing of onabotulinumtoxinA, and treatment outcomes in patients with CD. Despite years of use in thousands of patients and clinical evidence from randomized controlled trials, questions remain regarding the optimal use of onabotulinumtoxinA for CD. In disorders, such as CD, where there is variability in clinical presentation and severity of symptoms, as well as a variety of comorbidities and use of concomitant medications, optimal treatment schemas may not be available from controlled clinical trials.

Experience indicates that proper muscle selection and dose are key determinants for a good response. However, in current practice, there is lack of consensus regarding technical aspects of the use of onabotulinumtoxinA, such as the optimal dose, dilution ratios, number of injection sites, combination and number of muscles to inject, dosing interval, and targeting procedure [10-15]. Determinants that impact treatment decisions, such as disease severity and clinical presentation, have not been clearly identified. Furthermore, specialists involved in the treatment of CD, including neurologists and physiatrists, may use different approaches to treatment and injection techniques, for which the effect on outcomes is unknown. A number of studies have shown that CD adversely affects quality of life [4,16,17] and symptom relief following botulinum toxin treatment results in improvement [18-22]. However, many quality of life measures used in prior clinical trials were not disease-specific and were not sensitive enough to detect clinically relevant changes.

By capturing real-world treatment practices and patient outcomes, CD PROBE will attempt to answer a number of clinical questions noted above or, at minimum, generate reasonable hypotheses for further investigation, to optimize outcomes among CD patients being treated with onabotulinumtoxinA.

## Methods/Design

The primary objectives for CD PROBE, as predefined by the CD PROBE Charter Committee (JJ, MS, CA, PDC, CC, and MB) are to determine if:

• the presentation of anatomical subtypes of CD correlate with the Toronto Western Spasmodic Torticollis Rating Scale (TWSTRS) scores and global assessment of severity rating;

• specific presentations of CD inform treatment choices (muscles injected, number of sites injected, dose, and dilution);

• there are clinically definable severity subtypes (e.g. mild, moderate, severe) that correlate with CD scales/questionnaires;

• a new pictorial version of a scale to assess disease severity, the Pictorial Spasmodic Torticollis Rating Scale (P-STRS), correlates with other measures and is sensitive to change with treatment;

• the impact of disease and treatment affects quality of life;

• there are potential predictors of outcomes, including baseline presentation, treatment approach, injector's practice characteristics, and adverse effects.

### The CD PROBE Study

CD PROBE is a multicenter, national, prospective, standard-of-care, observational clinical registry designed to capture real-world clinical practices of neurologists, physiatrists, and other physicians who regularly treat CD. Including a range of clinical practices will allow comparison of treatment patterns for CD between physician groups having a wide range of experience in treating CD.

### Subjects

This prospective clinical registry includes subjects with a diagnosis of CD, who in the clinical judgment of the investigator, are considered to be candidates for onabotulinumtoxinA therapy and/or who are new to the physician's practice or new to botulinum toxin therapy. Subjects who had previously participated in a clinical trial using botulinum toxin may be included if the time since their last dose is greater than 16 weeks. Subjects excluded are those planning elective surgery during the study; women who are nursing, pregnant, or planning a pregnancy; subjects with a history of poor compliance with medical treatment; and any condition or situation which, in the physician's opinion, could place the subject at risk, confound the registry data, or interfere significantly with the subject's participation in the registry. Institutional Review Board approval was granted at each participating site and written informed consent will be obtained from all patients prior to any study procedures being performed.

Baseline information collected includes demographics; history of CD diagnosis; and past treatments, comorbidities, and medications, including over-the-counter medications. Although CD presents with mixed symptoms, the CD classification of the predominant feature (anterocollis, lateralcollis, retrocollis, or torticollis) and the predominant direction of pull is recorded. Information is also collected that characterizes the physicians' practice and their experience with botulinum toxin (Table 1).

**Table 1:** Information collected to characterize the treating physician's practice site and experience in using botulinum toxin for CD

Physician specialty	○ Neurologist ○ Physical medicine and rehabilitation ○ General practitioner ○ Pain specialist
Type of practice	○ Private, academic, health maintenance organization, general neurology, movement disorders focused ○ Total number of patients seen per week ○ Number of CD patients per week ○ Total number of CD patients in practice

Experience in practice and with botulinum toxin for CD	○ Years of treating CD with botulinum toxin ○ Clinical research experience with botulinum toxin ○ Formal botulinum toxin injection training ○ Years in practice ○ Board certification

CD: cervical dystonia.

The aim of this study is to gain valuable information on the current treatment of CD with various patient types and different clinical practices, which will require a large number of subjects to be enrolled. With 600 patients enrolled as of February 2011, it is anticipated that more than 1,000 patients will be enrolled by the end of 2011.

### Visits and assessments

**Figure 1:**
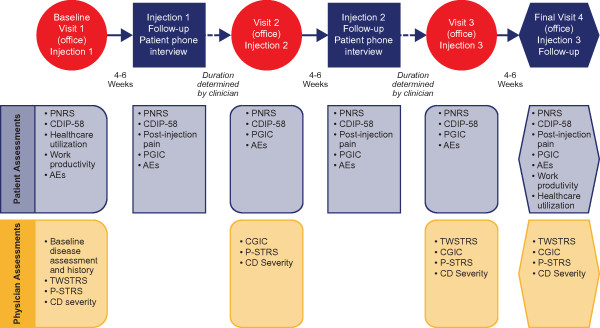
**CD PROBE study design and assessment of physician-reported and patient-reported outcome measures**. CD: cervical dystonia; PNRS: Pain Numeric Rating Scale; CDIP-58: Cervical Dystonia Impact Profile; AE: adverse event; PGIC: Patient Global Impression of Change; TWSTRS: Toronto Western Spasmodic Torticollis Rating Scale; P-STRS: Pictorial Spasmodic Torticollis Rating Scale; CGIC: Clinician Global Impression of Change.

The study period includes 3 injection cycles of onabotulinumtoxinA. Subjects are evaluated for safety and efficacy at each injection and at the peak effect 4 to 6 weeks following the injection. Follow-up visits after the first and second injections are via a telephone interview and follow-up after the third injection at the physician's office (Figure 1). Baseline data are collected prior to the first injection. The dosing and injection pattern are those customary for the practice of the physician. Information is collected on the dilution of onabotulinumtoxinA, dosing, use of electromyography, and muscles injected.

A comprehensive evaluation of outcomes is made by physicians and subjects using questionnaires that assess disease-specific outcomes, severity and improvement of symptoms, impact on overall well-being, quality of life, and impact of CD on work productivity and utilization of healthcare resources (Figure 1). The assessments that are made by the physician include the following:

1. TWSTRS is a validated, disease-specific questionnaire that comprises 3 subscales assessing severity (0 to 35), disability (0 to 30), and pain (0 to 20), including a total score (0 to 85) [23]. It is the most commonly used outcome measure in clinical trials of CD and botulinum toxins [9].

2. P-STRS is a new, disease-specific tool being developed to assess CD severity. Based on the TWSTRS severity subscale, P-STRS uses pictorial representations of the anatomical head and neck position. Preliminary assessments indicate that it is a valid and reliable tool that is sensitive to symptom improvement following treatment [24].

3. Clinician Global Impression of Change (CGIC) is a general questionnaire that provides the physician's assessment of the global impression of change using a 7-point scale ranging from "very much improved" to "very much worse" [25]. This questionnaire is used to determine if the change is clinically meaningful.

4. CD severity rating is the physician's assessment of the severity (mild, moderate, or severe) of the symptoms directly related to CD. This assessment establishes clinically relevant "cut points" with questionnaires that may help physicians in optimizing treatment with onabotulinumtoxinA based on the severity of symptoms.

Patient-reported outcomes are assessed using a variety of questionnaires that evaluate symptoms and the impact of CD-associated symptoms on their daily living and are as follows:

1. Pain Numeric Rating Scale (PNRS) is a single-item questionnaire of the current level of pain on a scale from 0 to 10 [26].

2. Post-injection pain questionnaire is a 2-item questionnaire that assesses if neck pain was relieved during the month after onabotulinumtoxinA injection. If some pain relief occurred, subjects are asked to provide the number of days after injection before they experienced relief of neck pain.

3. Patient Global Impression of Change (PGIC) is a general questionnaire that assesses the change in health status since the start of the study using a 7-point scale ranging from "very much improved" to "very much worse." It provides insight into the clinical value of the treatment [27].

4. Cervical Dystonia Impact Profile (CDIP-58) is a validated, disease-specific questionnaire that measures quality of life using 58 items that form 8 distinct subscales (head and neck, pain and discomfort, upper limb activities, walking, sleep, annoyance, mood, and psychological). It is more sensitive in detecting statistical and clinical change than comparable subscales of the SF-36, Functional Disability Scale, and Pain and Activities of Daily Living subscales of the TWSTRS [20,28].

5. Healthcare utilization questionnaire was developed for this study and is used to assess the use of healthcare resources (doctor and allied healthcare visits, emergency department visits, and hospitalizations) for treatment of CD symptoms.

6. Work productivity questionnaire prospectively elicits information regarding employment status, effect of CD on employment and productivity, and impact of treatment in restoring employment status [29].

Subjects who do not achieve any relief in symptoms 1 month after the first injection are assessed for a cause of the lack of response, according to routine medical practice, and then appropriateness of therapy, site of injection, dosage, and other factors are re-evaluated. The unilateral brow injection testing or serum antibody testing can be used to assess if toxin neutralizing antibodies are the cause of the lack of response [30]. Subjects are withdrawn from the study if they fail the brow test or if neutralizing antibodies are present.

Safety and tolerability are documented at each visit, with notation of the occurrence of adverse events, date of onset and resolution (if applicable), severity (mild, moderate, or severe), duration, frequency, relationship to study treatment, remedial actions, and outcome.

### Statistical analysis plan

The CD PROBE study design is a clinical registry; therefore, there is no prespecified hypothesis. Descriptive statistics and exploratory analyses of baseline and post-treatment outcome data will be performed. All analyses will be performed using R version 2.12.1 or greater (The R Foundation for Statistical Computing; http://www.r-project.org/).

#### Baseline results

As of February 4, 2011, 613 subjects have been enrolled into CD PROBE from 76 centers across the United States. Of the 77 principal investigators who have enrolled patients into CD PROBE, 68 are neurologists, 8 are physical medicine and rehabilitation specialists, and 1 is a pain specialist. Baseline demographics and disease characteristics have been tabulated for 609 subjects and are shown in Table 2. Most subjects (63.7%) were botulinum toxin-naïve and only 2.5% had previously received other forms of treatment (8 surgical denervation, 5 deep brain stimulation, 1 phenol injection, and 1 muscle resection surgery). Baseline information regarding the impact of having CD on work productivity is shown in Table 3.

**Table 2:** Subject baseline demographics and disease characteristics

Characteristic (n = 609)*	n (%) or mean ± SD (range)
Gender, female	462 (75.9)

Age, y	57.6 ± 14.3 (19.4-90.2)

Race/Ethnicity	
White	570 (93.6)
Hispanic	17 (2.8)
Asian	11 (1.8)
Black	9 (1.5)
Native American	1 (0.2)
Other	1 (0.2)

BMI, kg/m^2 ^	26.4 ± 5.4

Age at CD symptom onset, y (n = 608)	48.3 ± 16.2 (0.0-89.3)

Time from CD onset to CD diagnosis, y (n = 608)	5.4 ± 8.6 (-0.3-53.7)

Time to CD treatment after diagnosis, y (n = 608)	1.0 ± 3.5 (-0.3-31.4)

Total TWSTRS (n = 604)	38.2 ± 13.2 (4-077.0)

*Number of patients with data was 609 unless otherwise specified. SD: standard deviation; BMI: body mass index; CD: cervical dystonia; TWSTRS: Toronto Western Spasmodic Torticollis Rating Scale.

**Table 3:** Work productivity assessment of patients at baseline

Work productivity assessment	No. of patients	Response, n (%) or mean ± SD
Employed at baseline	575	Yes: 262 (45.6)

Employed when CD symptoms began	313	Yes: 161 (51.4)

Stopped working due to CD	161	Yes: 59 (36.6)

Employment status affected by CD	262	• Different job with less responsibility or pay: 14 (5.3)
		• Same job, reduced hours or responsibility: 50 (19.1)
		• No change: 198 (75.6)

Missed work in past month due to CD	261	Yes: 74 (28.4)

Number of missed work days in past month	74	5.7 ± 11.6

Decreased productivity due to CD	261	Yes: 150 (57.5)

Estimated decrease in work productivity (%)	150	72.1 ± 20.5

Have received disability benefits due to CD	262	Yes: 12 (4.6)

Duration of disability benefits (months)	12	33.2 ± 60.8

CD: cervical dystonia; SD: standard deviation.

## Discussion

OnabotulinumtoxinA is a safe and effective treatment for CD that improves the quality of life of sufferers [20-22]. Multiple outcome measures, including those assessed by patients and physicians, have been used to demonstrate effectiveness. In CD PROBE, comprehensive questionnaires, clinical rating scales, and other assessments are used to optimize the information collected. This large clinical registry of prospectively followed patients will allow analyses of determinants of outcome and adverse effects. Although onabotulinumtoxinA has been shown to be generally well tolerated with an acceptable adverse event profile, the occurrence of troublesome side effects, such as muscular weakness and dysphagia, and more systemic side effects, such as occasional malaise and other flu-like symptoms [31], may potentially lead to considerable disability and discontinuation of further treatment. Risk factors for the occurrence of adverse effects will be evaluated.

The baseline demographics and clinical characteristics of the CD PROBE population suggest that this cohort is representative of the general population with CD [32,33]. As previously reported [34-38], symptoms of CD typically emerge in the fifth decade of life and the disorder is more frequent in women than in men. In our population of 613 CD subjects, 63.7% of whom were never treated with botulinum toxin, the mean age at symptom onset was 48.3 ± 16.2 years, and the average duration of symptoms was 5.4 ± 8.6 years. The disabling nature of CD is supported by the findings that 36.6% stopped working because of CD, 28.4% missed work because of CD in the past month (the average number of days missed was 5.7 days), and 57.5% felt that their productivity was decreased due to CD (Table 3).

Large clinical registries, such as CD PROBE, can inform clinical practice by providing substantial amounts of data; however, they also have inherent limitations. The lack of a controlled design and a prespecified statistical analysis plan leaves the ability to answer clinical questions with statistical power to chance; therefore, large numbers of subjects are needed. By not controlling the inclusion/exclusion of patients in clinical registries, it is more likely that subjects with comorbid conditions will be included compared with those typically enrolled in controlled trials. Nonetheless, clinical registries may provide data on the clinical nuances of treatment that are not generally obtainable from randomized controlled trials and, therefore, are more representative of the "real-world" experience.

CD PROBE is anticipated to provide clinically relevant information about disease-specific outcomes following treatment with onabotulinumtoxinA, including reduction in pain, change in disease severity, CD-related quality of life, impact of treatment on overall health, and economic aspects of CD, including healthcare utilization and work productivity. The data generated from the study is anticipated to answer a number of questions regarding the optimal use of onabotulinumtoxinA for CD or generate hypotheses for investigation, with the ultimate goal to provide refinements in treatment to further improve outcomes in CD treatment.

## Competing interests

JJ: Baylor College of Medicine receives income from grants and contracts with Allergan, Inc., Ipsen, and Merz Pharmaceuticals for research led by Dr Jankovic. Dr Jankovic is a paid consultant to or a board member of Allergan, Inc., EMD Serono, Inc., Lundbeck Inc., Merz Pharmaceuticals, Michael J Fox Foundation for Parkinson's Research, Teva Pharmaceutical Industries Ltd, and receives payments/royalties from Elsevier, MedLink, *Neurology*, *Neurology in Clinical Practice*, Neurotoxin Institute, Scientiae, and UpToDate, Inc.

CHA: Received consulting fees from Allergan, Inc., Eli Lilly and Company, Ipsen, Medtronic, and Merck Serono.

PDC: Vanderbilt University receives income from grants and contracts with Allergan, Inc. and Medtronic for research led by Dr Charles. Dr Charles receives income from Allergan, Inc., Medtronic, and Pfizer Inc for education and consulting services.

M Stacy: Received compensation from Allergan, Inc., Boehringer Ingelheim, General Electric, Novartis, Osmotica Pharmaceutical Corp., Synosia Therapeutics, Schering-Plough, GlaxoSmithKline, Teva Pharmaceutical Industries Ltd, Biogen Idec, and Neurologix, Inc. for consulting, speaker bureau, protocol steering committee, and/or safety monitoring boards. Dr Stacy received royalties from Informa Press and research support from Ceregene, Inc., IMPAX, Michael J Fox Foundation for Parkinson's Reasearch, Neuraltus Pharmaceuticals, Inc., Novartis, Parkinson Study Group, and Schering-Plough.

M Schwartz: Employee of MedNet Solutions, Inc. who is contracted with Allergan, Inc. for data collection and statistical analysis.

SMS: Employed by UBC-Envision Group who contracted with Allergan, Inc. to provide professional writing assistance and project management support in the development of this manuscript.

MFB: Employed by Allergan, Inc. and holds Allergan stock.

SP: Employed by Allergan, Inc. and holds Allergan stock.

## Authors' contributions

JJ was involved in the conception and design of the study, acquisition of data, analysis and interpretation of data, drafting all or part of the manuscript, critical revision of the manuscript for intellectual content, and overall supervision as first author. CHA was involved in the conception and design of the study, analysis and interpretation of data, and critical revision of the manuscript for intellectual content. PDC was involved with analysis and interpretation of data, critical revision of the manuscript for intellectual content, and supervision. M Stacy was involved in the conception and design of the study, critical revision of the manuscript for intellectual content, and supervision. M Schwartz, the statistician, was involved with conception and design of the study, acquisition of data, analysis and interpretation of data, drafting all or part of the manuscript, and statistical expertise. SMS was involved with acquisition of data, drafting all or part of the manuscript, administration, and provided technical support. MFB was involved in the conception and design of the study, analysis and interpretation of data, critical revision of the manuscript for intellectual content, obtaining funding, and supervision. SP was involved with conception and design of the study, acquisition of data, analysis and interpretation of data, drafting all or part of the manuscript, critical revision of the manuscript for intellectual content, obtaining funding, administrative and technical support, and supervision. All authors have read and approved the final manuscript.

## Pre-publication history

The pre-publication history for this paper can be accessed here:

http://www.biomedcentral.com/1471-2377/11/140/prepub
